# Alternative splicing of CERS2 promotes cell proliferation and migration in luminal B subtype breast cancer cells

**DOI:** 10.18632/oncoscience.531

**Published:** 2021-04-14

**Authors:** Trishna Pani, Kajal Rajput, Animesh Kar, Ujjaini Dasgupta

**Affiliations:** ^1^Amity Institute of Integrative Sciences and Health, Amity University Haryana, Panchgaon, Manesar, Gurgaon 122413, Haryana, India; ^2^Laboratory of Nanotechnology and Chemical Biology, Regional Centre for Biotechnology, NCR Biotech Science Cluster, Faridabad 121001, Haryana, India

**Keywords:** breast cancer, alternative splicing, ceramide synthase 2

Breast cancer is broadly categorised into four different clinical subtypes, Luminal A, Luminal B, Triple-negative (TNBC), and HER2+ based on histological and molecular characterizations, distinct gene expression profiles and alternative splicing patterns that impact the choice and response to treatment [1]. Based on the unique molecular portrait, among the Estrogen receptor (ER) positive and responsive subtypes, Luminal A manifests less aggressive cell proliferation, better response to endocrine therapy, lower lung and liver metastatic rate, and higher recurrence free survival (RFS) compared to Luminal B subtype. In contrast, ER-negative TNBC and HER2^+^ subtypes show poor prognosis, diverse resistance patterns to different cytotoxic agents, high invasion and metastasis to brain, lung or visceral organs, and significantly reduced overall survival [2]. Therefore, each of these subtypes need a robust array of dependable histopathological and molecular markers that can be exploited to predict prognosis, and response to treatment.

Sphingolipids are a group of bioactive lipids, well-known for their diverse signalling roles in cellular regulatory circuits including inflammation, migration, proliferation, apoptosis and multiple drug resistance. All sphingolipids have a sphingosine backbone with varied polar headgroups and variable fatty-acyl chains with their functional diversity contingent to the carbon chain length and degree of saturation. The most prominent sphingolipids like ceramides, ceramide-1-phosphate, sphingosine-1-phosphate, and glucosylceramides have been reported to modulate different phenotypic hallmarks of cancer like proliferation, invasion, and migration [3]. Alteration in expression of sphingolipid metabolic enzymes affect the lipid homeostasis leading to dysregulation of the cellular signalling pathways involved in oncogenesis. Differential sphingolipid gene expression patterns are reported for each subtype that contribute to their distinct phenotypes [4]. Microarray analysis has shown that enzymes like *SPHK1, UGT8, ST8SIA1* are upregulated in ER-negative tumors whereas ER-positive tumors showed an increase in expression of *UGCG, CERS4, CERS6, ASAH1* [5]. In recent years, alternative splicing (AS) has emerged as a crucial post-transcriptional regulatory mechanism as the relative ratio of different splice isoforms of genes reveal imbalance in their abundance, many of which were not evident by conventional gene expression analysis alone [6]. The differentially expressed transcript isoforms of various oncogenic or growth pathways are unique to each breast cancer subtype, and therefore have high potential to be developed as valuable targets for cancer therapy [7]. On the same ground, it is likely that the RNA splicing signature for genes of sphingolipid pathway are distinct for all breast cancer subtypes, an area less ventured till now.

To elucidate the role of AS in sphingolipid metabolizing genes in cancer, we used bioinformatic approach, and identified AS events in sphingolipid genes for different breast cancer subtypes from TCGA BRCA dataset. The AS signature for sphingolipid genes predicted a unique *ceramide synthase 2* (*CERS2*) cassette exon event for exon 8 in Luminal B breast cancer subtype generating an AS transcript that was not identified before. Ceramide, the metabolic hub of the sphingolipid pathway is an antiproliferative, proapoptotic tumor suppressor lipid. Mammalian *ceramide synthase* (*CERS*) has six isoforms and each of them synthesizes a distinct chain-length ceramide. Expression of *CERS2, CERS4* and *CERS6* are found to be high in malignant breast tumors [8]. *CERS2* is the only *CERS2* that synthesises very long chain ceramides (C22:0, C24:0, C24:1) involved in preventing cell invasion and metastasis by impairing the role of matrix metalloproteinases [9]. Exon 8 of CERS2, corresponds to almost the entire Lag1p motif that imparts acyl chain substrate specificity to the protein, and is a part of the TRAM, Lag1p and CLN8 (TLC) catalytic domain of CERS2 protein [10].

Using polysome profiling and LC-MS/MS analysis of the junction peptide (between exon 7 and 9) in the AS form, we proved that CERS2 AS protein is active and translated in BT-474, a representative cell line for Luminal B subtype. The elevated expression of AS transcript and the AS protein in Luminal B patient tumor samples as compared to matched adjoining normal tissue showed direct relevance to the role of AS protein in tumor progression. Survival analysis of Luminal B subtype patients of TCGA-BRCA cohort showed that the Exon 8 skipping event in *CERS2* predicts poor prognosis. To show the functional validation of the AS protein, we overexpressed the AS protein in BT-474 cells, and performed in vitro proliferation and migration assay. AS overexpressed BT-474 cells were not able to attenuate proliferation or migration of BT-474 cells as efficiently as the CERS2 protein coding (PC) transcript derived protein. Further, the CERS2 AS overexpressed BT-474 cell line showed significantly lower levels of very long-chain ceramides with no apparent changes in level of short- or long-chain ceramides.

Our findings validate the importance of a fine balance of different chain-length specific ceramides in maintenance of normal physiological functions of the cell that gets perturbed during tumor development. The potential of AS form of CERS2 as a marker for diagnosis of the disease and prediction for survival prognosis is also established. However, this needs to be validated in a larger patient cohort and in patients of other subtypes, as well as in other cancer types that will ensure the robustness and specificity of the event. The broader relevance of our findings elucidate the role of post-transcriptional regulatory events in sphingolipid genes as a contributor to the enigmatic area of lipid reprogramming in tumors. It is prudent to propose that many more such alternative splicing events in sphingolipid genes, as we predict, are contributors to alterations in the profile and function of lipids in tumors. Interestingly, the myriad of AS events in sphingolipid genes may possibly contribute to the high levels of sphingolipids like ceramides in tumor tissues, that opposes their antiproliferative role already established by in vitro and *in vivo* studies [11].

**Figure 1:**
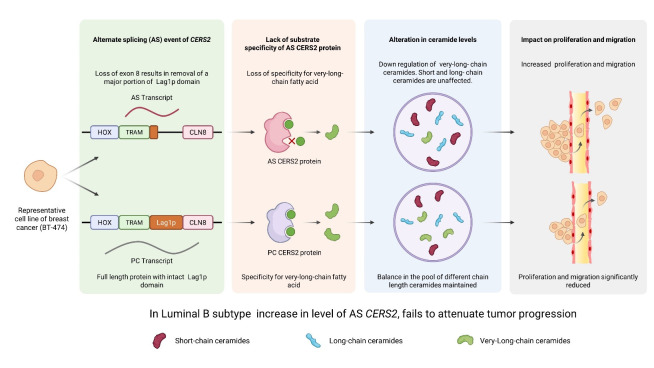
Figure 1: Schematic representation showing increased expression of AS CERS2 in Luminal B breast cancer subtype downregulates the level of very-long chain ceramides that fail to attenuate tumor growth and progression
